# Mutation at the Site of Hydroxylation in the Ribosomal Protein uL15 (RPL27a) Causes Specific Changes in the Repertoire of mRNAs Translated in Mammalian Cells

**DOI:** 10.3390/ijms24076173

**Published:** 2023-03-24

**Authors:** Elizaveta A. Zolotenkova, Alexander V. Gopanenko, Alexey E. Tupikin, Marsel R. Kabilov, Alexey A. Malygin

**Affiliations:** Institute of Chemical Biology and Fundamental Medicine, Siberian Branch of the Russian Academy of Sciences, Novosibirsk 630090, Russia

**Keywords:** HEK293T cells, RPL27a, uL15 hydroxylation, RNA-seq, polysome profiling, differentially expressed genes, translation

## Abstract

Ribosomal protein uL15 (RPL27a) carries a specific modification, hydroxylation, at the His39 residue, which neighbors the CCA terminus of the E-site-bound tRNA at the mammalian ribosome. Under hypoxia, the level of hydroxylation of this protein decreases. We transiently transfected HEK293T cells with constructs expressing wild-type uL15 or mutated uL15 (His39Ala) incapable of hydroxylation, and demonstrated that ribosomes containing both proteins are competent in translation. By applying RNA-seq to the total cellular and polysome-associated mRNAs, we identified differentially expressed genes (DEGs) in cells containing exogenous uL15 or its mutant form. Analyzing mRNA features of up- and down-regulated DEGs, we found an increase in the level of more abundant mRNAs and shorter CDSs in cells with uL15 mutant for both translated and total cellular mRNAs. The level of longer and rarer mRNAs, on the contrary, decreased. Our data show how ribosome heterogeneity can change the composition of the translatome and transcriptome, depending on the properties of the translated mRNAs.

## 1. Introduction

The precise work of the translation machinery, which carries out the synthesis of new proteins in the cell, in accordance with the codon sequences of translated mRNAs, is determined by the correct structure of the ribosome [1,2,3,4,5]. The mammalian ribosome consists of four chains of rRNAs and 80 ribosomal proteins, some of which carry various post-translational modifications (PTMs) [4,6,7,8,9,10]. Ribosomal proteins uL2, uL15, and uS12 (previously named as L8, L27a, and S23, respectively), are hydroxylated in the ribosome, and contain this PTM at the Cβ atom of the specific His (uL2 and uL15) or Pro (uS12) residues [11]. Hydroxylation of these ribosomal proteins is carried out by specific 2-oxoglutarate (2OG)/ Fe(II)-dependent oxygenases, accordingly named as ribosomal oxygenases (ROXs) [9,12]. It is important to note that the above PTMs of all three proteins are located near the functional centers of the ribosome (see [11,13,14,15,16]), which suggests the importance of hydroxylation of these proteins for the correct translational activity of the ribosome. In particular, the hydroxylated His39 residue of uL15 is located in the vicinity of the CCA terminus of the E-site-bound tRNA, and is adjacent to the binding site of the translation inhibitor, cycloheximide [17]. 

The regulation of the level of ribosomal protein uL15 is closely associated with carcinogenesis. uL15 has been shown to be significantly upregulated in metastatic triple-negative breast cancer (TNBC) cells, and its overproduction potentially promoted the development and metastasis of TNBC via the EIF2 signaling pathway [18]. On the contrary, its down-regulation with participation of a pivotal miRNA regulator, miR-595, induces p53 activation, apoptosis and inhibits proliferation, and in patients with myelodysplastic syndrome (MDS) with a miR-595 gene loss mutation, the risk of MDS is increased [19]. Considering that the level of uL15 hydroxylation with specific ROX decreases during hypoxia [9], and cell hypoxia accompanies cancer progression [20,21], one can expect a relationship between a decrease in the level of hydroxylated L15 and the development of cancer. 

We have previously shown that exogenous FLAG-tagged uL15 carrying the His39Ala substitution, and therefore being incapable of hydroxylation, can successfully incorporate into 60S ribosomal subunits in HEK293 cells [22]. Nevertheless, the level of polysomes in cells producing the mutant form of uL15 was significantly reduced compared to cells containing the wild-type form of exogenous uL15 [22]. Thus, one can propose that specific disturbances in mRNA translation occur in cells with non-hydroxylated form of uL15 in the ribosomes. 

The next-generation sequencing (NGS) of RNA (RNA-seq) is currently an advanced method for studying changes in the transcriptome and translatome landscapes of cells in response to various stimuli, including the deficiency of individual ribosomal proteins [23,24,25,26,27] or the introduction of specific mutations in them [28]. Being applied to the analysis of total cellular mRNA and mRNAs translated in polysomes, this method makes it possible to draw conclusions concerning the regulation of expression of individual genes at the levels of transcription and translation, and to evaluate their translational efficiencies. Therefore, it would be promising to determine changes in the overall pattern of translated mRNAs in cells with ribosomes carrying non-hydroxylated uL15.

In this study, we investigate how the failure of uL15 hydroxylation affects the pools of the total and translated mRNAs in HEK293T cells. We show that the efficiency of translation in cells producing the exogenous uL15 with the H39A mutation is reduced compared to cells with the wild-type exogenous protein. Using RNA-seq and comparing the datasets obtained with the mutant and the wild-type proteins, we identify genes with altered expression (differently expressed genes, DEGs) at the total transcription and at the translation levels (tDEGs and pDEGs, respectively) depending on the form of exogenous uL15. By analyzing the features of mRNAs from the up- and down-regulated sets of tDEGs and pDEGs, we found more intense translation of shorter and more abundant mRNAs in cells with the uL15 mutant. 

## 2. Results

### 2.1. Incorporation of the Mutant uL15 Incapable of Hydroxylation into Ribosomes Reduces Translational Activity in HEK293T Cells

**Figure 1:**
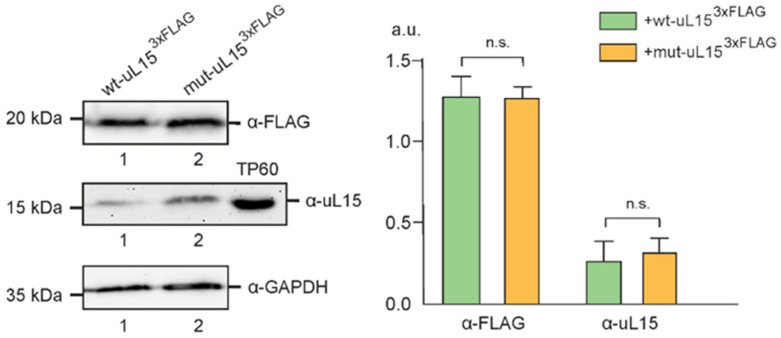
Production of exogenous 3×FLAG-tagged wild-type ribosomal protein uL15 (wt-uL15^3×FLAG^), and its mutated form with the H39A substitution (mut-uL15^3×FLAG^) in HEK293T cells, transfected by corresponding constructs. On the left, Western blot analyses of exogenous 3×FLAG-tagged proteins and endogenous uL15 and GAPDH as references in the cell lysates from wt-uL15^3×FLAG^-producing (1) and mut-uL15^3×FLAG^-producing (2) cells, using specific antibodies. Antibodies against natural uL15 did not react to exogenous forms of uL15, because of the 3×FLAG-tag interference. TP60, the total protein of 60S ribosomal subunits used as a reference for the position of endogenous uL15. On the right, a diagram showing the densitometry analysis of the Western blot data from three biological replicates of the experiment in arbitrary units (a.u.) as the mean±SEM. Note that there is no difference in the ratio of exogenous-to-endogenous uL15 signals between cells producing different FLAG-tagged proteins.

To produce the C-terminal 3×FLAG-tagged ribosomal protein uL15, carrying the H39A substitution at the specific hydroxylation site (mut-uL15^3×FLAG^) in HEK293T cells, the cells were transiently transfected with a plasmid construct bearing a minigene of this protein. Cells transfected with the same construct encoding the 3×FLAG-labeled wild-type protein (wt-uL15^3×FLAG^) were used as a control. The experiment was performed in three biological replicates. After 2 days of transfection, exogenous proteins were synthesized in the respective cells along with endogenous uL15 (Figure 1 and Figure S1).

**Figure 2:**
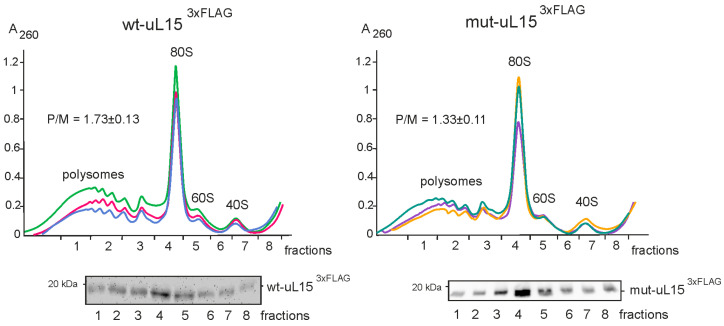
Polysome profiles obtained from the lysates of HEK293T cells, producing wt-uL15^3×FLAG^ (top left) and mut-uL15^3×FLAG^ (top right). Profiles from three biological replicates are presented in different colors and superimposed. Peaks corresponding to polysomes, 80S monosomes, and 60S and 40S ribosomal subunits are designated. The mean ratio of the polysome peaks to the monosome peak (P/M) with the SD, determined from biological replicates, is shown on each panel. Western blot analyses of the content of FLAG-labeled exogenous proteins in the gradient fractions are shown below the polysome profiles.

Cell lysates obtained from transfected cells were centrifuged in a linear 5–50% sucrose density gradient, and the resulted polysome profiles were fractionated. Analysis of the content of wt-uL15^3×FLAG^ and mut-uL15^3×FLAG^ in the gradient fractions by Western blotting showed that both proteins were present in all fractions containing 60S ribosomal subunits, both free and being a component of 80S monosomes and polysomes (Figure 2). This proved that 3×FLAG-tagged uL15 was able to replace its endogenous counterpart in the 60S ribosomal subunits and the resulting subunits retained their ability to participate in translation, and that the mutation H39A did not deprive ribosomes with the mutant mut-uL15^3×FLAG^ of the ability to participate in protein biosynthesis. However, the polysome/monosome ratio (P/M) in the polysome profiles with mutant protein was markedly lower than that in the profiles with the wild-type protein, indicating inefficient translation in cells where mut-uL15^3×FLAG^ replaces endogenous uL15 in ribosomes. This is consistent with the early data [22] and points to the importance of uL15 hydroxylation for the correct functioning of the ribosome. 

### 2.2. RNA-Seq of Total and Polysome-Associated mRNAs from Cells Producing Mut-uL15^3×FLAG^ or Its Wild-Type Form

To find changes in the cell’s transcriptome and translatome in response to the production of non-hydroxylated mut-uL15^3×FLAG^, we performed RNA-seq of both total mRNA isolated from transfected cells and polysome-associated mRNAs from the corresponding sucrose gradient fractions, respectively. The basic characteristics of the DNA libraries prepared from the resulting RNA samples are presented in Table S1 and Figures S2 and S3. The principal component analysis (PCA) evaluation of the primary RNA-seq data demonstrated a satisfactory degree of clustering between biological replicates (Figure S4), implying that the data obtained were applicable for the subsequent analysis.

**Figure 3:**
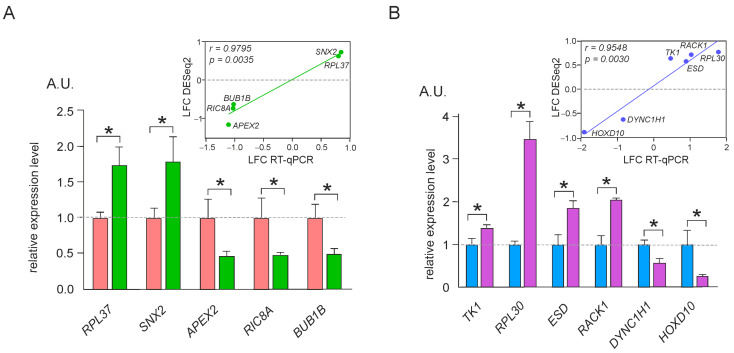
The validation of differential gene expression analysis by RT-qPCR. Data of the relative expression level for representative tDEGs (**A**) and pDEGs (**B**) in HEK293T cells, producing wt-uL15^3×FLAG^ and mut-uL15^3×FLAG^, are presented as the mean ± SEM of arbitrary units (A.U.) from three biological replicates (* *p* < 0.05, Mann–Whitney test). Green (**A**) and pink (**B**) columns show the relative levels of gene expression in cells producing mut-uL15^3×FLAG^ compared to the levels of gene expression in cells producing wt-uL15^3×FLAG^, which are shown in rose and blue columns, and taken as 1. The correlation between RT-qPCR and RNA-seq data is shown in the top right.

Analysis of differential gene expression performed for RNA-seq data with the use of the DESeq2 package made it possible to determine and compare the contents of transcriptomes and translatomes in cells producing mut-uL15^3×FLAG^ and wt-uL15^3×FLAG^ (Tables S2 and S3). To reveal sets of statistically significant differentially expressed genes (DEGs), cutoffs for the adjusted *p*-value (p.adj < 0.05), the absolute value of the shrunken Log2 fold-change (|LFC| > 0.322), and the base mean value (> 100) were applied. Hence, tDEGs were determined from the analysis of cellular transcriptomes and pDEGs were found from the similar analysis of translatomes (polysome-associated mRNAs). In these terms, tDEGs were the genes of which the content of total mRNA (both translated and non-translated) was altered in cells with mut-uL15^3×FLAG^, as compared to the cells with wt-uL15^3×FLAG^, whereas pDEGs were the genes of which the mRNA content was changed only in the polysomes. The total set of tDEGs included 122 down-regulated and 133 up-regulated genes, while the set of pDEGs consisted of 89 and 229 such genes, respectively (Table S4). To validate the results of the DEG analysis performed with the RNA-seq data, we carried out RT-qPCR for a representative group of DEGs. The values of changes in the expression of these genes, estimated by RT-qPCR, correlated well with the respective values obtained with the RNA-seq data analysis (Figure 3), indicating the reliability of the tDEGs and pDEGs found.

**Figure 4:**
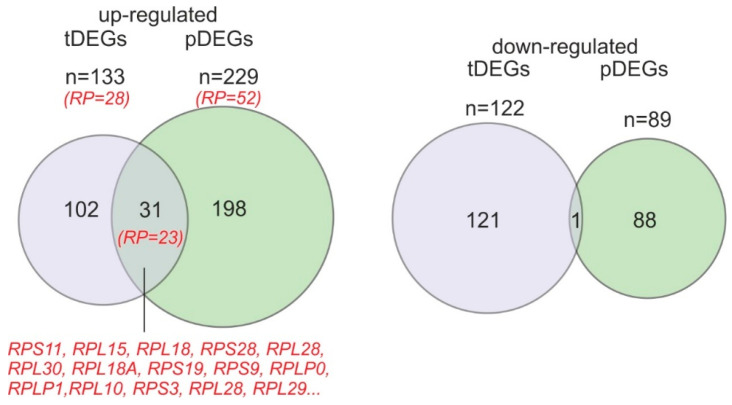
Venn diagrams showing the overlap of up-regulated and down-regulated tDEGs and pDEGs. Ribosomal protein genes are designated in red.

It is remarkable that quite a large part of the up-regulated tDEG and pDEG sets consisted of the cytosolic ribosome proteins genes, 28 and 53 genes, respectively, (e.g., *RPS29, RPL10, RPL30, RPS21, RPL9, RPL39, RPL18, RPS13, RPL23A, RPS25, RPS19*, and many others). In addition, a significant number of genes of the respiratory chain complex proteins was also observed in these sets, 8 and 7 genes, respectively, (such as *NDUFA1, NDUFA3, NDUFA4, ATP5F1E, NDUFS5, NDUFS7, NDUFB7, NDUFB9, ATP5ME, COX7C*, and others). It should also be noted that the sets of the up-regulated tDEGs and pDEGs overlapped by 31 genes, although at the same time, the sets of down-regulated tDEGs and pDEGs matched only in one gene, i.e., they had almost nothing in common (Figure 4). Moreover, the majority of the overlapped genes between up-regulated tDEG and pDEG sets were ribosomal protein genes (23 genes). Thus, the contents of the cell translatome and transcriptome change during the mut-uL15^3×FLAG^ synthesis in the cell. Since the set of pDEGs correlates rather poorly with the set of tDEGs, one can assume that the change in the composition of translated mRNAs occurs largely independently of the change in the transcriptome pattern.

### 2.3. Analysis of the tDEG and pDEG mRNAs

**Figure 5:**
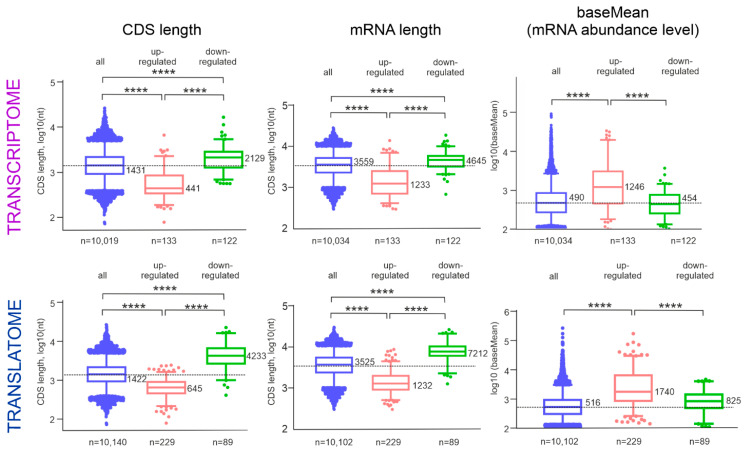
Length and abundance of the up-regulated and down-regulated DEG mRNAs. Data on the CDS and mRNA lengths and the mRNA abundance levels (baseMean) are presented in columns, and data for tDEGs and pDEGs are presented in rows. Each graph contains three box-and-whiskers plots showing the distribution of up-regulated tDEGs or pDEGs (rose), down-regulated tDEGs or pDEGs (green) and for all sequenced genes (blue) by the length of their CDSs or mRNAs, or by base mean. The boxes represent the 25th percentile, median (values are designated on the right of boxes), and 75th percentile, and whiskers indicate the 5–95% range; **** *p* < 0.0001.

To reveal the distinctive characteristics that have mRNAs of up- and down-regulated tDEGs and pDEGs, we analyzed the cellular abundance, length, and composition of the respective mRNAs. To estimate the relative abundance of each mRNA, we used the average of the normalized count values (baseMean) obtained in RNA-seq (Table S4). A comparison between mRNAs of up- and down-regulated DEGs showed that, on average, the relative abundances of the former considerably exceeded the relative abundances of the latter for both tDEGs and pDEGs (Figure 5). Additionally, the relative abundances of up-regulated mRNAs were, on average, higher than those for all cellular mRNAs as a whole, whereas the relative abundances of the down-regulated mRNAs were, on average, lower than those of mRNAs as a whole (Figure 5).

Next, for genes from the sets of up-regulated and down-regulated DEGs, we compared the total lengths of their mRNAs (Table S5) and the lengths of the coding sequences (CDSs) of these mRNAs (Table S6). It turned out that the mRNA lengths of the up-regulated tDEGs were shorter than those of all cellular mRNAs and significantly shorter than those of down-regulated tDEG mRNAs on average. The median length of down-regulated tDEG mRNAs was much longer than that of up-regulated tDEG mRNAs. The same was observed for the mRNA of pDEGs (Figure 5). Moreover, similar and even substantial differences between mRNAs of up- and down-regulated DEGs were also noticed when comparing the mRNA CDS lengths (Figure 5). A comparison between the GC content of the up-regulated and down-regulated DEG mRNAs did not reveal any significant difference between the mRNA sets (data are not presented).

Thus, we arrived to the conclusion that, in the presence of a pool of ribosomes with non-hydroxylated mutant uL15 in the cell, significant changes occur in the patterns of total cellular and translated mRNAs, together with a general decrease in the level of polysomes. Such a reorganization of the cell’s transcriptome and translatome is expressed in an increase in the population of shorter and more prevalent mRNAs, while the portion of long and less abundant mRNAs decreases. These changes are associated, most likely and predominantly, with disorders in the process of mRNA translation, rather than with the transcription of genes, since pDEGs would otherwise be highly dependent on tDEGs. In our opinion, the overlap of up-regulated tDEGs and pDEGs is explained by the fact that a change in the pool of translated mRNAs, especially in the case of highly abundant mRNAs, should also be noticeable in the general pool of cellular mRNAs, since the latter also includes translated mRNAs.

## 3. Discussion

In this work, using RNA-seq-based approaches, we showed how the substitution of His39, which is normally hydroxylated in ribosomal protein uL15, with alanine leads to changes in both the entire pool of cellular mRNAs and mRNAs translated by ribosomes. We found that the mutation His39Ala does not affect the participation of uL15 in the 60S ribosomal subunit assembly, but the appearance of the ribosomes containing 60S subunits with the mutated protein increases the translational level of the shorter and more abundantly represented mRNAs. This, in turn, leads to changes in both the translatome and the transcriptome as a whole. Such changes are noticeable in a significant increase in the expression level of many ribosomal protein genes, as well as of many genes of the respiratory chain complex proteins. These data clearly indicate the importance of the hydroxyl group at the His39 in ribosomal protein uL15 for both the operation of the ribosome machinery and the general level of translational in the cell. 

**Figure 6:**
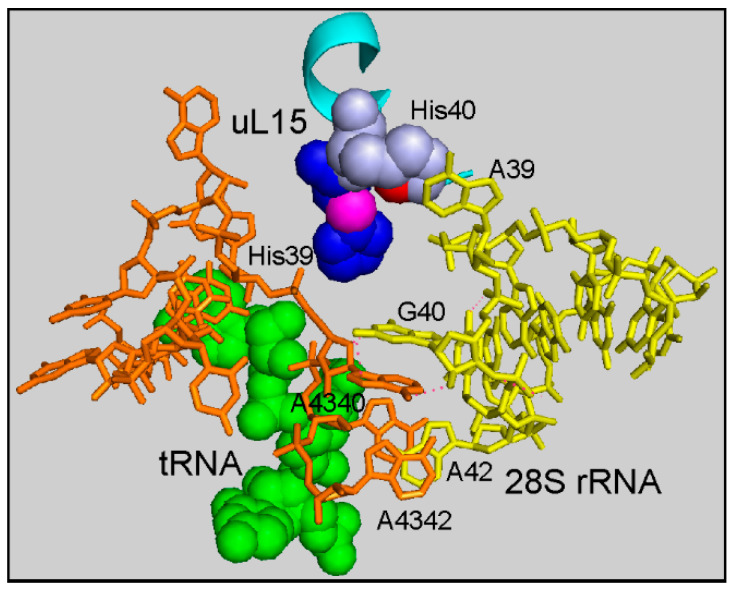
Fragment of the cryo-EM structure of the human ribosome around the residue His39 of uL15 (PDB: 6QZP, [29]). The nucleotides of the helices H11 and H82 of 28S rRNA are shown by sticks and are colored in yellow and orange, respectively. The CCA terminus of the E-site-bound tRNA is represented by green balls. Residues His39 and His40 of uL15 (cyan) are shown by blue and grey balls, respectively. The Cβ atom of His39 (magenta), which contains a hydroxyl group (not resolved in the structure), and the Nδ atom (red) of His40, which possibly forms a hydrogen bond with the hydroxyl group at Cβ, as hypothesized in [22], stabilize the protein structure. Note the position of His39 within the E-site-bound tRNA environment. Figure drawn in the PyMOL Molecular Graphics System software [30].

Why does the appearance of ribosomes with the His39Ala mutated protein uL15 in the pool of cellular ribosomes lead to an increase in the level of predominantly short and highly represented mRNAs? The hydroxylated His39 in uL15 is located far from the mRNA channel in the 80S ribosome and does not interact with mRNA, so it cannot affect the translation by selecting particular types of mRNAs. Most likely, the observed changes in the mRNA pool occur due to different activities of ribosomes with the mutant uL15, as compared to that with the wild-type protein. Residue His39 in uL15 is located in close proximity to the E site of the ribosome and is involved in the maintaining of the structural environment of the 3’-end of the E-site-bound tRNA (Figure 6).

It is noteworthy that the ribosome region under consideration also contains the binding site for the translation inhibitor cycloheximide [31,32]. This compound binds close to the site of the 3’-end of the tRNA residing in the E-site of the ribosome, in the pocket formed with the participation of 28S rRNA and proteins uL15 and eL42, which blocks translation [3,17]. Thus, the correct structural organization of the ribosome region neighboring the H39 residue in uL15 is extremely important for efficient translation elongation. Therefore, one can assume that the His39Ala mutation disturbs, albeit slightly, the structure of the E-site region, and thereby affects the elongation stage of translation, so that the uL15-mutant ribosomes translate mRNA less efficiently than those with the wild-type protein. 

In a situation where there are two types of ribosomes in a cell, with the hydroxylated uL15 protein and with its non-hydroxylated mutant form, which differ from each other in the efficiency of mRNA translation, translation elongation disorders may occur. On long mRNAs which are translated by a large number of ribosomes simultaneously, the frequency of such disorders should be higher than on short mRNAs. This reasoning can explain the changes in the pool of translated mRNAs in cells with non-hydroxylated mut-uL15^3×FLAG^ towards an increase in mRNAs with shorter CDSs to some extent. Thus, it is not the selection of mRNA by ribosomes with non-hydroxylated uL15, but likely the lower efficiency of these ribosomes that gives shorter and more abundant mRNAs an advantage in translation.

In addition, it should be taken into account that the cell must respond to changes in the gene expression and the resulting protein imbalance. Apparently, the inability of the mutant uL15 to be hydroxylated is perceived by the cell as a signal of oxygen deficiency, which causes increased expression of many genes involved in aerobic respiration and respiratory electron transport chain. Although mRNAs of these genes are not as highly represented in the cell as mRNAs of ribosomal proteins, they are nevertheless short enough (their CDS lengths are on average about 300 nt) to gain an advantage in translation in the presence of ribosomes with non-hydroxylated uL15. 

## 4. Materials and Methods

### 4.1. Plasmids, Cell Culturing, and Target Protein Production Analysis

The plasmids design for wt-uL15^3×FLAG^ and mut-uL15^3×FLAG^, cloning of the minigene of C-terminal 3×FLAG-tagged human ribosomal protein uL15 in vector pcDNA3.1 and its mutagenesis for the H39A codon substitution, were described in [22]. The HEK293T cells (CVCL_0063) were cultured in the 10 cm Petri dishes containing DMEM, 10% FBS, and 100 U/mL of penicillin–streptomycin, in a CO_2_ incubator (5% CO_2_) at 37°C. When reaching a confluence of 50–60%, the cells were transiently transfected with the above plasmids using the Turbofect transfection reagent (Thermo Fisher Scientific Waltham, MA, USA), according to the manufacturer’s protocol. After 48 h of incubation, the cells were incubated for 10 min in ice-cold phosphate-buffered saline (PBS) with cycloheximide (100 mcg/ml), then washed with PBS, followed by collection from the Petri dishes. The experiment was performed in four biological replicates.

One third of collected cells was lysed in Tryzol reagent (Ambion, Waltham, MA, USA), and total RNA was isolated for RNA-seq, according to the manufacturer’s protocol. The remaining cell portion was suspended for 10 min in ice-cold lysis buffer containing 20 mM Tris–HCl (pH 7.5), 15 mM MgCl_2_, 200 KCl, and 1% Triton X-100. The resulting lysate was centrifuged for 10 min at 14,000 g and 4°C, and the supernatant was layered on a linear gradient of 5–50% sucrose in 50 mM Tris–HCl (pH 7.5) buffer containing 100 mM KCl and 12 mM MgCl_2_. The sucrose gradient was centrifuged for 18 h at 17,000 g and 4°C in a SW-40 rotor, and fractionated as described in [23]. The content of the polysome fractions was precipitated with one volume of ice-cold EtOH and RNA from the pellet was isolated as in [23]. The integrated optical densities of the peaks of polysomes (P) and monosomes (M) were calculated from the file, with the readings of the UV absorption at 260 nm measured for 0.36 sec each in the flow cell of the Milichrom A-02 chromatograph (Econova, Novosibirsk, Russia), and the P/M ratio was calculated for each replicate.

The production of the target and reference proteins was confirmed by the Western blotting of small aliquots of lysates and gradient fractions. Each lane of the gel contained lysate material obtained from 300,000 cells, or half of the material precipitated from the gradient fraction with EtOH (see above). Mouse monoclonal antibodies specific for FLAG-peptide (M2, #F1804, Sigma, St. Louis, MO, USA) and GAPDH (#60004-1-Ig, ProteinTech, Singapore), and rabbit polyclonal antibodies against uL15 (RPL27A, #PA-5-45706, Invitrogen, Waltham, MA, USA) were used in the protein detection.

### 4.2. DNA Library Preparation and Next-Generation Sequencing

RNA extracted from samples of total cellular lysate and polysome gradient fractions were quality-checked with the Bioanalyzer 2100, using the RNA6000Pico kit (Agilent Technologies, Santa Clara, CA, USA), as described above. DNA libraries were prepared using the MGIEasy RNA Directional Library Prep Set (MGI Tech, Shenzhen, China) according to the manufacturer’s instructions, and subjected to the next-generation sequencing (NGS) on the MGIseq-2000 platform, utilizing the 2x100 PE sequencing mode (FCL PE100, MGI Tech). All relevant procedures were performed in the SB RAS Genomics Core Facility (ICBFM SB RAS, Novosibirsk, Russia). 

### 4.3. Raw RNA-Seq Data Processing

The quality of the raw reads in fastq formats was examined with the help of the FastQC (v.0.11.9) and MultiQC (v. 1.9) tools, and then subjected to the quality filtration (Trimmomatic 0.39) and adapter trimming (cutadapt 2.9), using adapter sequences provided by the manufacturer. The filtered reads were quality-assessed and mapped to the hg38 reference human genome using the STAR RNA-seq aligner tool (2.7.3) and the Ensembl annotation (release 102). The qualitative evaluation of the BAM files obtained was performed with the QualimapTool (v.2.2), using default parameters. The analysis of the gene coverage with the sequencing reads was obtained using the IGV genome browser and generated BAM files. The generated quality metrics were collected in a metadata table. The RNA-seq read data were submitted to the GenBank under the study accession number PRJNA932595.

### 4.4. Downstream Analysis of Processed RNA-Seq Data

The count table was obtained with the Rsubread (v. 2.10.5), using the GTF (ensemble release 104) as annotation in paired-end and reversely stranded mode. Batch effects were removed using the sva package (v. 3.44.0), according to batches described in the Table S7. The differential expression analysis was done by DESeq2 (v. 1.36.0) (p.adj cutoff < 0.05, shrunken LFC cutoff < 0.322 and > 0.322, base mean cutoff > 100). All metadata regarding the transcript features (CDS, 5’- and 3’-UTRs sequences, length, and GC-content) were collected with the biomaRt (v. 2.52.0) and calculated applying the Biostrings (v. 2.64.1), with the use of the latest Ensembl version (v. 108). Only the information regarding the main form of transcripts for each gene was collected by selecting the “canonical” flag. All the graphs, calculations, and statistical analyses were performed using the GraphPad Prism software v. 7.01. 

### 4.5. Validation of RNA-Seq Results Using RT-qPCR

In 20 µL of the reverse-transcription reaction, 2 µg of RNA isolated from corresponding samples was incubated with 100 pmol of random primer and 25 U of MMLV reverse-transcriptase, according to the manufacturer’s protocol (Thermo Fischer Scientific, Waltham, MA, USA). The cDNAs obtained were used in the qPCR experiments that were performed on the LightCycler 96 (Roche, Basel, Switzerland), with the SYTO9 fluorescent dye (Thermo Fischer Scientific, Waltham, MA, USA), hot-start HS-Taq polymerase (Biolabmix, Novosibirsk, Russia), and gene-specific primers (Table S8). The experiments were carried out for 3 biological replicates and in 2–3 technical replicates. The parameters of qPCR were as follows: 95 °C for 30 s, 45 cycles 95 °C for 10 s, 55 °C for 20 s, and 72 °C for 20 s (single acquisition). The relative quantification of gene expression levels was calculated using the integrated LightCycler 96 (Roche, Basel, Switzerland) software, with the genes encoding GAPDH and 18S rRNA used as references.

## 5. Conclusions

Our studies of changes in the transcriptome and translatome of HEK293T cells, caused by an elimination of the hydroxylation site in the ribosomal protein uL15, revealed significant rearrangements in the balance of cellular mRNAs. We showed that in cells with the above protein mutation, the composition of translated mRNAs shifts towards shorter and more abundant mRNAs, while longer and rarer mRNAs are translated poorly. We attributed these changes to the fact that ribosomes that do not contain hydroxylated His39 in uL15 translate mRNA inefficiently and interfere with ribosomes containing the wild-type uL15. Similar processes may occur in cancer cells undergoing hypoxia, and therefore possibly having an increased portion of non-hydroxylated uL15. Further research in this direction will help shed light on the role of the hydroxyl group on His39 in the structure of the ribosome and in the molecular mechanics of translation.

## Data Availability

The RNA-seq read data were submitted to the GenBank under the study accession number PRJNA932595.

## Supplementary Materials

The supporting information can be downloaded at: https://www.mdpi.com/article/10.3390/ijms24076173/s1.
