# Supplementary material for: Mutation at the Site of Hydroxylation in the Ribosomal Protein uL15 (RPL27a) Causes Specific Changes in the Repertoire of mRNAs Translated in Mammalian Cells
Source: Int J Mol Sci. 2023 Mar 24;24(7):6173. doi: 10.3390/ijms24076173 (PMC10094517; doi:10.3390/ijms24076173)
Supplement: Supplementary file 1 [file ijms-24-06173-s001.zip › Supplementary materials.pdf]

Supplementary materials

Figures

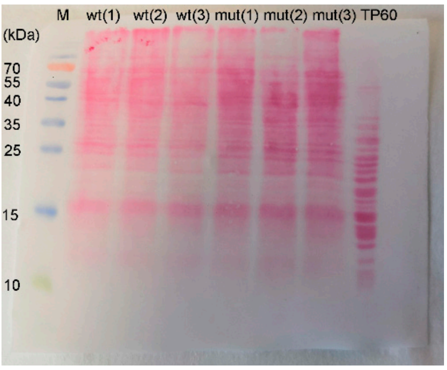

Figure S1. Stained membrane after separating cell lysate proteins in 15% SDS-GAGE and transferring them to nitrocellulose. Lanes wt(1-3) and mut(1-3), lysates from cells producing wt-uL15$^{3\times FLAG}$ and wt-uL15$^{3\times FLAG}$, respectively, in three biological replicates. TP60, the total protein of 60S ribosomal subunits. M, the PageRuler Prestained Protein Ladder (Thermo Scientific, #26616) as protein weight marker with indication of the protein's masses. This membrane was used in Western blot experiments to identify proteins of interest.

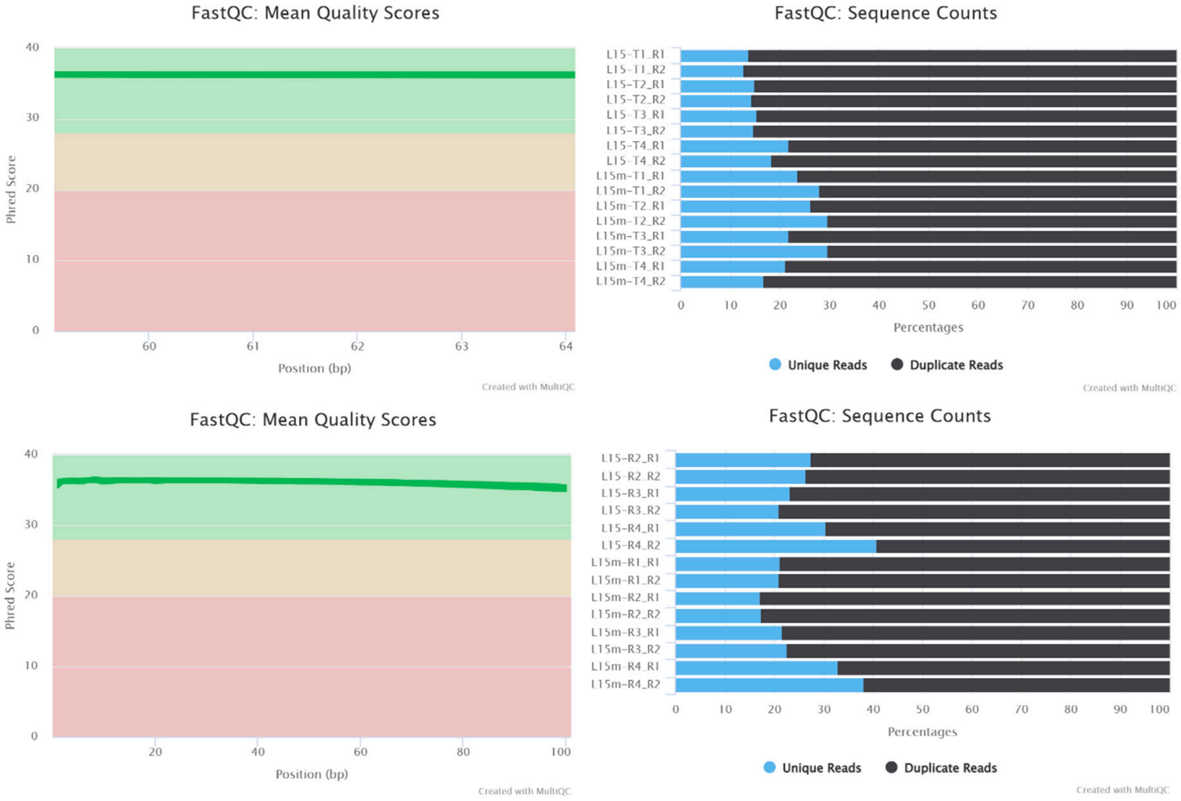

Figure S2. The raw data quality metrics obtained for sequenced libraries with the FastQC and summarized with the MultiQC tools. Upper panels, mean quality scores and duplicate rates for the RNA-seq data obtained with total mRNA; bottom panels, the same with the polysome-associated mRNA. Indexing of libraries: L15 and L15m are for cells produced wt-uL15$^{3\times FLAG}$ and mut-uL15$^{3\times FLAG}$, respectively; T1-T4 and R1-R4 are for total and polysome-associated mRNA.

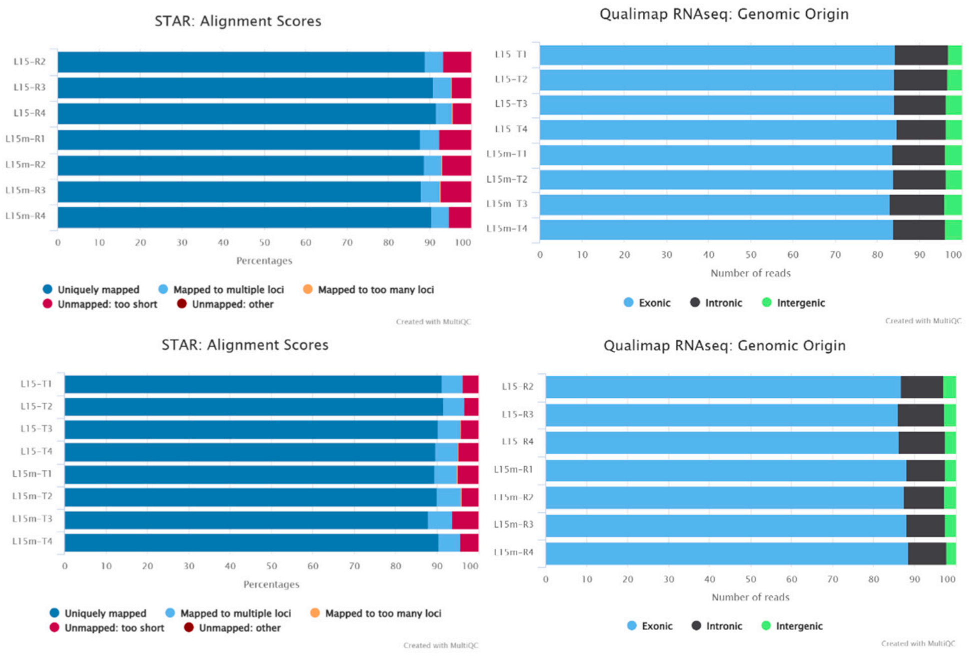

Figure S3. General mapping statistics for the RNA-seq data with total and polysome-associated mRNA fractions. The QC reports are summarized by MultiQC based on STAR-generated mapping reports and Qulimap2 RNA-seq QC reports. Data for the libraries generated from polysome-associated and total mRNA are presented on the upper and bottom panels, respectively. Indexing of libraries as above.

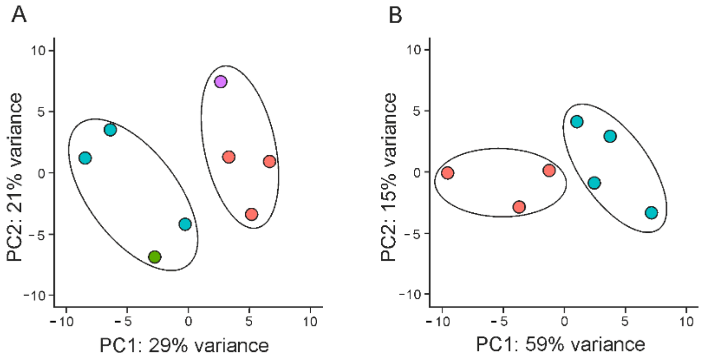

Figure S4. Principal components analysis (PCA) for RNA-seq obtained with the total mRNA (A) and polysome-associated mRNA (B) samples showing the clustering between biological replicates of cells produced wt-uL15$^{3\times FLAG}$ or mut-uL15$^{3\times FLAG}$.

Tables

Table S1. Basic characteristics of the sequencing libraries and mapped reads (indexing of libraries as above).

Sample Name	5'-3' bias	M Aligned
**L15-T1**	1.18	10.6
**L15-T2**	1.19	10.5
**L15-T3**	1.19	12.0
**L15-T4**	1.27	33.2
**L15m-T1**	1.17	11.0
**L15m-T2**	1.18	4.8
**L15m-T3**	1.17	15.0
**L15m-T4**	1.19	38.9
**L15-R2**	1.21	7.8

**L15-R3**	1.16	8.6
**L15-R4**	1.23	30.7
**L15m-R1**	1.21	12.3
**L15m-R2**	1.23	12.9
**L15m-R3**	1.15	13.5
**L15m-R4**	1.23	22.1

Table S2. The DESeq2 metadata of the differential expression analysis of the RNA-seq data obtained with total RNA from cells produced wt-uL15$^{3\times FLAG}$ or mut-uL15$^{3\times FLAG}$ (Excel table).

Table S3. The metadata of the differential expression analysis of the RNA-seq data obtained with polysome-associated mRNA from cells produced wt-uL15$^{3\times FLAG}$ or mut-uL15$^{3\times FLAG}$ (Excel table).

Table S4. The subsets of up-regulated and down-regulated tDEGs and pDEGs obtained from the data of Tables S2 and S3 by filtering (cutoffs: padj < 0.05, |LFC Shrunken| > 0.322, baseMean > 100) (Excel table).

Table S5. mRNA lengths for up-regulated and down regulated tDEGs and pDEGs, and for all genes sequenced from total and polysome-associated mRNA with baseMean > 100 (Excel table).

Table S6. CDS lengths for up-regulated and down regulated tDEGs and pDEGs, and for all genes sequenced from total and polysome-associated mRNA with baseMean > 100 (Excel table).

Table S7. The samples batch effect used in the RNA-seq data analysis.

run	assay	condition	batch
L15-R2	PS	WT	I
L15-R3	PS	WT	I
L15-R4	PS	WT	II
L15m-R1	PS	H39A	I
L15m-R2	PS	H39A	I
L15m-R3	PS	H39A	I
L15m-R4	PS	H39A	II
L15-T1	RS	WT	I
L15-T2	RS	WT	I
L15-T3	RS	WT	I
L15-T4	RS	WT	II
L15m-T1	RS	H39A	I
L15m-T2	RS	H39A	I
L15m-T3	RS	H39A	I
L15m-T4	RS	H39A	II

Table S8. The list of oligonucleotide primers used for RT-qPCR.

Gene	Forward primer	Reverse primer
DYNC1H1	5’–ataagctcctaactttgccc-3’	5’–ttgttgaagatcatgtcggt-3’
ESD	5’–cgtggctgcaatattaaagg-3’	5’–attggcatttatgagttggg-3’
HOXD10	5’–ctccttcaccaccaacatta-3’	5’–ctaaaatatccagggacggg-3’
RACK1	5’ – aaacacctttacacgctaga -3’	5’ – ttgctgctggtactgataac -3’
RPL30	5’–ctctaggctccaactcgtta-3’	5’–gtgatggacaccagttttag-3’
TK1	5’–gtgcctggtgatcaagtatg-3’	5’–cgatgtcagggaaaaactgc-3’
APEX2	5’–gtcatagacacctttcaggc-3’	5’–ctaggaagcgaaggatcttg-3’
BUB1	5’–gcttcactgatagctgtacc-3’	5’ – gagaattcccctactcctgc -3’
RIC8	5’–tgtgtgatgatcgctactg-3’	5’–cgcttctctaggaagatgag-3’
RPL37	5’–ctaaggcctaccaccttca-3’	5’–ttgggtttaggtgttgttcc-3’
SNX2	5’–agagcagttaatacacaggc-3’	5’–ctatgacagaccaaggcttc-3’
